# Superiority of laparoscopic liver resection to open liver resection in obese individuals with hepatocellular carcinoma: A retrospective study

**DOI:** 10.1002/ags3.12506

**Published:** 2021-09-16

**Authors:** Atsushi Ishihara, Shogo Tanaka, Hiroji Shinkawa, Hisako Yoshida, Shigekazu Takemura, Ryosuke Amano, Kenjiro Kimura, Go Ohira, Kohei Nishio, Shoji Kubo

**Affiliations:** ^1^ Department of Hepato‐Biliary‐Pancreatic Surgery Osaka City University Graduate School of Medicine Osaka Japan; ^2^ Department of Medical Statistics Osaka City University Graduate School of Medicine Osaka Japan

**Keywords:** body mass index, hepatectomy, laparoscopic liver resection, obesity, postoperative complication

## Abstract

**Aim:**

This study aimed to elucidate the effects of laparoscopic liver resection (LLR) vs open liver resection (OLR) for major complications (Clavien‐Dindo classification grade ≥ IIIa) in obese individuals with hepatocellular carcinoma (HCC).

**Methods:**

The clinical records of 339 and 733 patients who underwent LLR and OLR, respectively, for HCC between 2000 and 2019 were retrospectively reviewed. Body mass index (BMI) groups were classified according to the definitions of the World Health Organization: underweight group, BMI ≤ 18.4 kg/m^2^ (LLR vs OLR: 27 vs 47); normal weight, BMI 18.5‐24.9 kg/m^2^ (211 vs 483); overweight, BMI 25.0‐29.9 kg/m^2^ (85 vs 181); and obese, BMI ≥ 30.0 kg/m^2^ (16 vs 22). The effects of obesity on major complications after LLR and OLR were investigated.

**Results:**

In total, 18 (5.3%) and 127 (17.3%) patients presented with major complications after LLR and OLR, respectively. There was no significant difference in the incidence of major complications after OLR in the four BMI groups. However, a stepwise decrease in the incidence of major complications after LLR was observed from the underweight to the obese group. In addition, a multivariate analysis revealed that increased BMI was an independent preventive factor for major complications after LLR (*P* = .026, odds ratio: 0.84). The estimated adjusted risk of major postoperative complications decreased with increased BMI in the LLR group, while the risk did not decrease in the OLR group (*P* for interaction = .048).

**Conclusion:**

Laparoscopic liver resection is beneficial for obese patients and is superior to OLR.

## Graphical abstract

We compared the effects of being overweight and obese on major complications after laparoscopic liver resection (LLR) and open liver resection (OLR) in patients with hepatocellular carcinoma. The estimated risk of postoperative major complications indicated downward trend in LLR according to increase in body mass index with significant interaction for OLR.

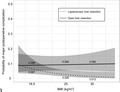

## 1 INTRODUCTION

The prevalence of obesity and its associated diseases is still increasing worldwide. The prevalence of obesity (body mass index [BMI] of ≥30 kg/m^2^) is 40% in the United States^1^ and approximately 20% in Europe.^2^ In Japan, obesity is defined by a BMI of ≥25 kg/m^2^.^3^ As of 2018, 32.2% of men and 21.9% of women of ≥20 years of age were classified as obese.^4^ Obesity is correlated with comorbidities and technical difficulties in surgery and is considered a risk factor for postoperative complications in several surgical fields.^5^, ^6^ Furthermore, several reports have shown that obese patients are at high risk of developing hepatocellular carcinoma (HCC).^7^, ^8^ Thus, a higher prevalence of obesity and expansion of the indications for liver resection could increase the number of liver resection procedures among obese patients with HCC in the future. Obesity is associated with an increased risk of postoperative morbidity in individuals undergoing open liver resection (OLR).^9^, ^10^ Recently, laparoscopic liver resection (LLR) has been widely performed and is correlated with low morbidity and mortality.^11^, ^12^ However, the superiority of LLR to OLR was not evaluated according to BMI. Thus, previous reports cannot support the efficacy and safety of LLR for obese individuals.^13^, ^14^

The current study investigated the effects of obesity on major complications (≥grade IIIa based on the Clavien‐Dindo classification system^15^) after LLR and OLR for HCC based on BMI (from underweight to obese). Moreover, the superiority of LLR to OLR in terms of major postoperative complications based on BMI was evaluated.

## 2 MATERIALS AND METHODS

### 2.1 Study design and participants

In total, 1072 consecutive patients with HCC who underwent liver resection in our department between January 2000 and December 2019 were included in this study. Patient's height and weight were assessed preoperatively, and BMI was calculated as weight in kilograms (kg) divided by height in meters squared (m^2^). The patients were allocated to one of four groups based on BMI, as defined by the World Health Organization^16^: underweight group, BMI of ≤18.4 kg/m^2^; normal weight group, 18.5 ≤ BMI ≤ 24.9 kg/m^2^; overweight group, 25.0 ≤ BMI ≤ 29.9 kg/m^2^; and obesity group, BMI of ≥30.0 kg/m^2^. The local institutional review board of our institution approved this study (registration no. 1646).

### 2.2 Surgical procedure

In total, 923 and 149 patients who underwent their first and second hepatic procedure, respectively, were included in the analysis. LLR was performed on 339 patients (LLR group) and OLR on 733 patients (OLR group). As described in our previous study on OLR,^17^ in most patients who underwent segmentectomy or more, after Glissonean sheath transection or clamping, an ultrasonic surgical aspirator was used for hepatic dissection during total or unilateral clamping of the hepatic vascular inflow. In the majority of patients who underwent partial hepatic resection, as resection of less than a segmentectomy, an ultrasonic surgical aspirator and bipolar or monopolar forceps was utilized for hepatic dissection with the Pringle maneuver. The major branches of the Glissonean sheath and the hepatic vein were sutured using non‐absorbent sutures. Patients who underwent LLR were placed in supine or left‐lateral decubitus position and on an average five trocars according to tumor location were used. Hepatic transection was performed using a laparoscopic ultrasonic surgical aspirator and a vessel sealing system with soft coagulation.^18^ In general, the Pringle maneuver was applied. Hand‐assisted laparoscopy, or the so‐called hybrid procedure or laparoscopy‐assisted resection, was performed on patients with tumors that are challenging to evaluate via pure laparoscopy due to limited visualization and heavy bleeding. In this study, LLR was defined as all laparoscopic surgeries. Further, the surgical procedures were classified into partial resection, segmentectomy, sectionectomy, and resection of two or more sections according to the Brisbane 2000 Terminology of Liver Anatomy and Resections.^19^

### 2.3 Indication for laparoscopic liver resection

We performed LLR for ≤5‐cm solitary lesions located in the peripheral liver segments 2‐6 according to the Louisville consensus.^20^ Thereafter, we extended the indication for more difficult procedures including major hepatectomy. However, LLR was selected according to tumor location, types of operative procedures, tumor size, proximity to major vessels, and liver function.

### 2.4 Clinicopathological characteristics and surgical outcomes

The clinical data of all patients were collected prospectively, as shown in Table 1. The date of follow‐up was on March 31, 2020.

**TABLE 1:** Clinicopathological characteristics and surgical outcomes of laparoscopic and open liver resection in patients with hepatocellular carcinoma (n = 1072)

Variables	Study cohort	Laparoscopic liver resection group	Open liver resection group	*P* value
	(n = 1072)	(n = 339)	(n = 733)	
BMI, kg/m^2^; median (range)	23.2 (12.4‐40.2)	23.6 (12.4‐40.2)	23.0 (15.0‐38.2)	.080
BMI groups, underweight/normal/overweight/obesity, n	74/694/266/38	27/211/85/16	47/483/181/22	.357
Age, years; median (range)	69 (19‐87)	70 (21‐87)	69 (19‐87)	.288
Sex, male/female, n	832/240	242/97	590/143	.001
Comorbidities and/or previous medical history, n (%)				
Diabetes mellitus	351 (32.7)	121 (35.7)	230 (31.4)	.162
Hypertension	525 (49.9)	183 (54.0)	342 (46.7)	.026
Dyslipidemia	196 (18.3)	79 (23.3)	117 (16.0)	.004
Ischemic heart diseases	51 (4.8)	15 (4.4)	36 (4.9)	.728
Alcohol abuse (≥60 g/d), n (%)	317 (29.6)	109 (32.2)	317 (43.2)	.208
Underlying hepatic disease, n (%)				
HBV	177 (16.5)	66 (19.5)	111 (15.1)	.248
HCV	572 (53.4)	180 (53.1)	392 (53.5)	
HBV + HCV	11 (1.0)	4 (1.2)	7 (0.9)	
Non‐B, non‐C	310 (28.9)	89 (26.3)	223 (30.4)	
Pathologically confirmed cirrhosis	324 (30.2)	91 (26.8)	233 (31.8)	.101
Laboratory tests				
Total bilirubin level, mg/dL; median (range)	0.7 (0.7‐2.7)	0.6 (0.2‐2.3)	0.7 (0.1‐2.7)	<.001
Albumin level, g/dL; median (range)	4.0 (2.3‐5.3)	4.1 (2.3‐5.3)	3.9 (2.6‐5.0)	<.001
Prothrombin time, %; median (range)	93 (13‐147)	93 (57‐144)	94 (13‐147)	.816
Child‐Pugh score, A/B, n	1035/37	710/23	325/14	.408
Platelet count, ×10^4^/µL; median (range)	14.8 (1.3‐46.2)	14.0 (4.0‐35.5)	15.1 (1.3‐46.2)	<.001
AST level, IU/L; median (range)	37 (11‐201)	32 (11‐201)	39 (12‐187)	<.001
ALT level, IU/L; median (range)	31 (5‐270)	26 (6‐166)	34 (5‐270)	<.001
Surgery‐related factors, n (%)				
Recurrence	277 (25.8)	95 (28.0)	182 (24.8)	.267
Repeat liver resection	149 (13.9)	43 (12.7)	106 (14.5)	.434
Types of liver resection				
Partial resection	669 (62.4)	278 (82.0)	391 (53.3)	<.001
Segmentectomy	98 (9.1)	14 (4.1)	84 (11.5)	
Sectionectomy	170 (15.9)	34 (10.0)	136 (18.6)	
Resection of two or more sections	135 (12.6)	13 (3.8)	122 (16.6)	
Conversion, n (%)	5 (0.5)	5 (1.5)		‐
Operative time, min; median (range)	276 (75‐915)	259 (75‐750)	276 (75‐915)	<.001
Volume of blood loss, mL; median (range)	340 (0‐7460)	100 (0‐3025)	500 (0‐7460)	<.001
Non‐curative surgery	25 (2.3)	2 (0.6)	23 (3.1)	.008
Tumor‐related factors				
AFP level, ≥ 20 ng/mL; n (%)	412 (38.4)	114 (33.6)	298 (40.7)	.028
Tumor size, cm; median (range)	2.7 (0.4‐21.0)	2.2 (0.4‐9.5)	3.1 (0.5‐21.0)	
Number, solitary/multiple; n (%)	798/274	286/53	512/221	<.001
Macrovascular invasion, n (%)	118 (11.0)	17 (5.0)	101 (13.8)	<.001
UICC stage, n (%)				
Ia	274 (25.6)	130 (38.3)	144 (19.6)	<.001
Ib	461 (43.0)	146 (43.1)	315 (43.0)	
II	246 (22.9)	60 (17.7)	186 (25.4)	
IIIa	44 (4.1)	1 (0.3)	43 (5.9)	
IIIb	42 (3.9)	2 (0.6)	40 (5.5)	
IVa	1 (0.1)	0	1 (0.1)	
IVb	4 (0.4)	0	4 (0.5)	
Pathology				
Poor HCC, n (%)	267 (24.9)	53 (15.6)	214 (29.1)	<.001
Number, solitary/multiple; n (%)	765/307	274/65	491/242	<.001
Microvascular invasion, n (%)	337 (31.4)	76 (22.4)	261 (35.6)	<.001
Postoperative complications, n (%)				
Overall	329 (30.7)	59 (17.4)	270 (36.8)	<.001
Major^†^	145 (13.5)	18 (5.3)	127 (17.3)	<.001
In‐hospital death	4 (0.4)	0	4 (0.5)	.173
Hospital stay, days; median (range)	13 (4‐212)	9 (4‐88)	15 (5‐212)	<.001

Abbreviations: AFP, alpha fetoprotein; ALT, alanine aminotransferase; AST, aspartate aminotransferase; BMI, body mass index; COPD, chronic obstructive pulmonary disease; HBV, hepatitis B virus; HCC, hepatocellular carcinoma; HCV, hepatitis C virus; UICC, Union for International Cancer Control.

^†^≥Grade IIIa based on the Clavien‐Dindo classification system.

### 2.5 Definitions

Patients were diagnosed with diabetes mellitus (DM), hypertension, and dyslipidemia according to the guidelines of the Japan Diabetes Society,^21^ Japanese Society of Hypertension,^22^ and Japan Atherosclerosis Society,^23^ respectively. DM was defined as a fasting plasma glucose level of ≥126 mg/dL, hemoglobin A1c level of ≥6.5%, or need for hypoglycemic drugs or insulin. Hypertension was defined as a systolic blood pressure of ≥140 mmHg, diastolic blood pressure of ≥90 mmHg, or need for antihypertensive drugs. Dyslipidemia was defined as a serum low‐density lipoprotein cholesterol level of ≥140 mg/dL, high‐density lipoprotein cholesterol level of <40 mg/dL, and/or triglyceride level of ≥150 mg/dL or a need for lipid‐lowering drugs. Regarding postoperative complications, wound infection was defined as the presence of bacteria in the wound exudate. Bile leakage was defined as a bilirubin concentration of at least three times the serum bilirubin concentration in the drainage fluid on or after postoperative day (POD) 3 or a need for radiological or surgical intervention for biliary collection or bile peritonitis.^24^ Intractable ascites was defined as drainage of 1 L/day for more than 2 days or ascites in the whole abdomen. Intractable pleural effusion was diagnosed when thoracentesis was performed.^25^ Pneumonia and atelectasis were considered respiratory complications.^26^ Liver failure was defined as the presence of prolonged hyperbilirubinemia (total serum bilirubin concentration of ≤3.0 mg/dL) on or after POD 5 and a need for fresh frozen plasma to decrease prothrombin time (≤50%) based on the modified definitions proposed by the International Study Group of Liver Surgery^27^ and Balzan et al.^28^ The severity of postoperative complications was graded according to the Clavien‐Dindo classification system.^15^ In this study, major complication was defined as ≥grade IIIa based on the Clavien‐Dindo classification system.

### 2.6 Outcomes

The primary outcome was the effect of BMI on major complications after LLR and OLR. Patients who underwent LLR and OLR initially presented with risk factors for major complications. Finally, to evaluate the superiority of LLR to OLR in patients with increased BMI, the difference in major complications between patients who underwent LLR and OLR was analyzed via a multivariate analysis.

### 2.7 Statistical analyses

Categorical variables were presented as numbers and percentages and were compared between groups using the Fisher's exact test or the *χ* ^2^ test, as appropriate. Continuous variables were expressed as median (range) and were compared using the Kruskal‐Wallis test. The Holm's method^29^ was used to adjust *P* values for multiple comparisons of demographic variables among the different groups. We performed the Cochran‐Armitage trend test^30^ to assess the categorical variables and the Jonckheere‐Terpstra trend test^31^ to assess the continuous variables. Univariate and multivariate logistic regression analyses were performed to evaluate the relative risk for major postoperative complications. A nonlinear restricted cubic spline was contained to consider the nonlinear effect of BMI on the risk for major postoperative complications. All statistical inferences were assessed using a two‐sided significance level of 5%, except for the interaction (cross‐product term) analysis. Variables with a *P* value of <.05 in the univariate analysis (Cox's proportional hazard model) were included in the multivariate analysis. A *P* value <.05 was considered statistically significant. All statistical analyses were performed using the Statistical Package for the Social Sciences® software version 26.0 (IBM Corp.) and EZR (Saitama Medical Center, Jichi Medical University, Saitama, Japan), which is a graphic user interface for the R software version 3.5.1 (The R Foundation for Statistical Computing).^32^

## 3 RESULTS

### 3.1 Characteristics of patients who underwent laparoscopic liver resection and open liver resection

There was no significant difference in terms of median BMI among patients who underwent LLR and OLR (Table 1). Among the patients who underwent LLR/OLR, 27/47, 211/483, 85/181, and 16/22 patients were classified into the underweight, normal weight, overweight, and obese groups, respectively. In the LLR group, the proportion of female patients, patients with HT, and patients with DL was higher when compared with the OLR group. The preoperative serum concentrations of total bilirubin, albumin, aspartate aminotransferase (AST), and alanine aminotransferase (ALT) were better in the LLR group than in the OLR group. However, the distribution of the Child‐Pugh scores among the two groups did not significantly differ. The proportion of patients who underwent partial liver resection was higher in the LLR group than in the OLR group, who had a shorter operative time and lower volume of blood loss. Based on tumor‐related factors or pathology, the OLR group had a more advanced HCC than the LLR group. The rate of incidence of overall and major postoperative complications was lower in the LLR group than in the OLR group, who had a shorter hospital stay.

### 3.2 Clinical characteristics of patients who underwent LLR and OLR according to BMI status

The clinicopathological characteristics of patients (n = 1072) after LLR or OLR according to BMI are shown in Table 2. In the LLR group, the proportion of patients with DM had a stepwise increase from the underweight to obese group. However, there was no significant difference in terms of other background characteristics, liver function test results, and tumor‐related factors among the four BMI groups. Moreover, type of liver resection, operative time, and volume of blood loss did not differ among the four groups. However, the conversion rate from laparoscopic to open surgery was high in the underweight group (*P* = .014). That is, the procedure was converted to open surgery in two (7.4%) patients in the underweight group because bleeding could not be controlled due to a narrow working space and in two patients in the normal weight and overweight groups because of injury in the major hepatic vein. Moreover, in one (6.3%) patient in the obesity group, the surgery was converted because of cancer invasion to the diaphragm (n = 1). By contrast, in the OLR group, the proportion of patients with DM, hypertension, dyslipidemia, and non‐B non‐C hepatitis indicated a stepwise increase from the underweight to obesity groups. There was a significant difference in the serum concentrations of albumin, AST, and ALT. However, the distribution of Child‐Pugh scores did not remarkably differ among the four groups. Although there was no difference in the type of liver resection among the four BMI groups, the proportion of patients who experienced bleeding indicated a stepwise increase from the underweight to obesity groups. In each BMI group, the proportion of patients who had intraoperative bleeding was higher in the OLR group than in the LLR group (*P* = .011 in the underweight group and *P* < .001 in the normal weight, overweight, and obesity groups).

**TABLE 2:** Clinicopathological characteristics of patients with hepatocellular carcinoma (n = 1072) after laparoscopic or open liver resection according to body mass index

Variables	Laparoscopic liver resection group							Open liver resection group					
	Underweight group	Normal weight group	Overweight group	Obese group	*P* value	*P* value for trend		Underweight group	Normal weight group	Overweight group	Obese group	*P* value	
	(n = 27)	(n = 211)	(n = 85)	(n = 16)				(n = 47)	(n = 483)	(n = 181)	(n = 22)		*P* value for trend
BMI, kg/m^2^; median (range)	17.4 (12.4‐18.4)	22.6 (18.5‐24.9)	26.9 (25.0‐29.7)	33.0 (30.1‐40.2)	<.001	<.001		17.4 (15.0‐18.4)	22.1 (18.5‐24.9)	26.5 (25.0‐29.9)	31.2 (30.0‐38.1)	<.001	<.001
Age, years; median (range)	70 (46‐83)	71 (21‐85)	71 (47‐87)	65 (44‐73)	.093	72 (19‐82)	69 (20‐87)	69 (37‐85)	70 (44‐78)	0.299	
Sex, male/female, n	14/13	153/58	63/22	12/4	.133	34/13	398/85	144/37	14/8	0.066	
Comorbidities and/or previous history, n (%)													
Diabetes mellitus	7 (25.9)	72 (34.1)	31 (36.5)	11 (68.8)	.029	.019		9 (19.1)	138 (28.6)	69 (38.1)	14 (63.6)	<.001	<.001
Hypertension	11 (40.7)	111 (52.6)	53 (62.4)	8 (50.0)	.206			11 (23.4)	213 (44.1)	102 (56.4)	16 (72.7)	<.001	<.001
Dyslipidemia	4 (14.8)	46 (21.8)	24 (28.2)	5 (31.3)	.380			2 (4.3)	65 (13.5)	45 (24.9)	5 (22.7)	<.001	<.001
Ischemic heart disease	0	11 (5.2)	4 (4.7)	0	.509			1 (2.1)	25 (5.2)	10 (5.5)	0	0.545	
Alcohol abuse (≥ 60 g/d), n (%)	7 (25.9)	70 (33.2)	25 (29.4)	7 (43.8)	.602			10 (21.3)	135 (28.0)	56 (30.9)	7 (31.8)	0.589	
Underlying hepatic diseases, n (%)													
HBV	6 (22.2)	39 (18.5)	17 (20.0)	4 (25.0)	.412			8 (17.0)	72 (14.9)	28 (15.5)	3 (13.6)	<.001	<.001
HCV	15 (55.6)	116 (55.0)	45 (52.9)	4 (25.0)				31 (66.0)	278 (57.6)	80 (44.2)	3 (13.6)		
HBV + HCV	0	4 (1.9)	0	0				1 (2.1)	4 (0.8)	2 (1.1)	0		
Non‐B, non‐C	6 (22.2)	52 (24.6)	23 (27.1)	6 (37.5)				7 (14.9)	129 (26.7)	71 (39.2)	16 (72.7)		
Pathologically confirmed cirrhosis	7 (25.9)	48 (22.7)	31 (36.5)	5 (31.3)	.112			15 (31.9)	143 (29.6)	64 (35.4)	11 (50.0)	.139	
Laboratory tests													
Total bilirubin level, mg/dL; median (range)	0.6 (0.3‐1.5)	0.6 (0.2‐2.2)	0.6 (0.2‐2.3)	0.8 (0.3‐2.3)	.124			0.6 (0.2‐1.4)	0.7 (0.1‐2.7)	0.7 (0.1‐2.2)	0.6 (0.2‐2.0)	.4	.041
Albumin level, g/dL; median (range)	4.1 (3.4‐5.3)	4.1 (2.3‐5.1)	4.2 (3.1‐4.8)	4.0 (3.6‐4.5)	.940			3.9 (3.2‐4.6)	3.9 (2.6‐5.0)	4.0 (2.1‐5.0)	3.7 (2.9‐4.3)	<.001	
Prothrombin time, %; median (range)	100 (71‐122)	93 (62‐144)	93 (57‐138)	89 (69‐114)	.083			91 (67‐123)	93 (13‐141)	94 (40‐130)	95 (71‐147)	.856	
Child‐Pugh score, A/B; n (%)	27/0	202/9	82/3	14/2	.253			46/1	464/19	179/2	21/1	.288	
Platelet count, ×10^4^/µL; median (range)	13.6 (6.0‐25.2)	13.6 (4.0‐35.5)	14.9 (4.1‐32.1)	15.3 (5.7‐29.7)	.488			16.0 (7.9‐34.0)	14.9 (2.2‐42.8)	15.3 (1.3‐46.2)	16.4 (1.7‐31.8)	.854	
AST level, IU/L; median (range)	34 (23‐201)	32 (11‐159)	32 (15‐163)	39 (11‐98)	.107			45 (14‐123)	40 (12‐187)	35 (13‐105)	38 (14‐110)	.005	<.001
ALT level, IU/L; median (range)	24 (13‐96)	26 (6‐166)	28 (8‐162)	34 (13‐91)	.478			32 (6‐113)	37 (8‐270)	31 (5‐172)	28 (11‐104)	.037	.022
Surgery‐related factors, n (%)													
Recurrence	8 (29.6)	60 (28.4)	22 (25.9)	5 (31.3)	.955			12 (25.5)	115 (23.8)	53 (29.3)	2 (9.1)	.163	
Repeat liver resection	3 (11.1)	30 (14.2)	10 (11.8)	0	.408			7 (14.9)	65 (13.5)	32 (17.7)	2 (9.1)	.488	
Types of liver resection													
Partial resection	21 (77.8)	166 (78.7)	77 (90.6)	14 (87.5)	.279		27 (57.4)		249 (51.6)	103 (56.9)	12 (54.5)	.548	
Segmentectomy	2 (7.4)	11 (5.2)	0 (0.0)	1 (6.3)			5 (10.6)		63 (13.0)	15 (8.3)	1 (4.5)		
Sectionectomy	4 (14.8)	24 (11.4)	5 (5.9)	1 (6.3)			8 (17.0)		84 (17.4)	39 (21.5)	5 (22.7)		
Resection of two or more sections	0	10 (4.7)	3 (3.5)	0			7 (14.9)		87 (18.0)	24 (13.3)	4 (18.2)		
Conversion, n (%)	2 (7.4)	1 (0.5)	1 (1.2)	1 (6.3)	.014	.826							
Operative time, min; median (range)	284 (135‐605)	253 (75‐750)	262 (87‐704)	270 (90‐593)	.614			271 (93‐610)	276 (75‐915)	276 (84‐662)	345 (105‐640)	.028	.062
Volume of blood loss, mL; median (range)	70 (0‐1850)	90 (0‐1625)	100 (5‐3025)	130 (10‐1540)	.247	.147		330 (10‐5100)	490 (0‐7460)	550 (0‐5050)	1230 (90‐6265)	.001	.002
Non‐curative surgery	0	2 (0.9)	0	0	.748			1 (2.1)	16 (3.3)	4 (2.2)	2 (9.1)	.35	
Tumor‐related factors													
AFP level, ≥ 20 ng/mL, n (%)	10 (37.0)	75 (35.5)	25 (29.4)	4 (25.0)	.637			21 (44.7)	197 (40.8)	69 (38.1)	11 (50.0)	.66	
Tumor size, cm; median (range)	2.0 (0.7‐7.8)	2.1 (0.4‐7.0)	2.5 (0.7‐9.0)	2.6 (1.7‐9.5)	.075			2.7 (0.6‐15.0)	3.0 (0.7‐20.0)	3.1 (0.5‐21.0)	5.1 (2.2‐12.0)	.004	.104
Number, solitary/multiple; n (%)	24/3	174/37	72/13	16/0	.265			35/12	337/146	124/57	16/6	.869	
Macrovascular invasion, n (%)	1 (3.7)	13 (6.2)	3 (3.5)	0	.590			6 (12.8)	67 (13.9)	22 (12.2)	6 (27.3)	.282	
UICC stage, n (%)													
Ia	14 (51.9)	86 (40.8)	27 (27.8)	3 (18.8)	.135			11 (23.4)	100 (20.7)	33 (18.2)	0	.245	
Ib	10 (37.0)	81 (38.4)	42 (42.4)	13 (81.3)				21 (44.7)	201 (41.6)	80 (44.2)	13 (59.1)		
II	3 (11.1)	41 (19.4)	16 (16.8)	0				9 (19.1)	124 (25.7)	49 (27.1)	4 (18.2)		
IIIa	0	1 (0.5)	0	0				3 (6.4)	26 (5.4)	12 (6.6)	2 (9.1)		
IIIb	0	2 (0.9)	0	0				3 (6.4)	30 (6.2)	5 (2.8)	2 (9.1)		
IVa	0	0	0	0				0	1 (0.2)	0 (0.0)	0		
IVb	0	0	0	0				0	1 (0.2)	2 (1.1)	1 (4.5)		
Pathology									(0.0)				
Poor HCC, n (%)	4 (14.8)	33 (15.6)	13 (13.3)	3 (18.8)	.987			16 (34.0)	137 (28.4)	52 (28.7)	9 (40.9)	.537	
Number, solitary/multiple; n (%)	24/3	164/47	70/15	16/0	.095			33/14	325/158	117/64	16/6	.797	
Microvascular invasion, n (%)	4 (14.8)	54 (25.6)	16 (18.8)	2 (12.5)	.301			17 (36.2)	178 (36.9)	58 (32.0)	8 (36.4)	.719	

Abbreviations: AFP, alpha fetoprotein; AST, aspartate aminotransferase; ALT, alanine aminotransferase; BMI, body mass index; COPD, chronic obstructive pulmonary disease; HBV, hepatitis B virus; HCC, hepatocellular carcinoma; HCV, hepatitis C virus; UICC, Union for International Cancer Control.

### 3.3 Major complications after LLR and OLR according to BMI status

**FIGURE 1:**
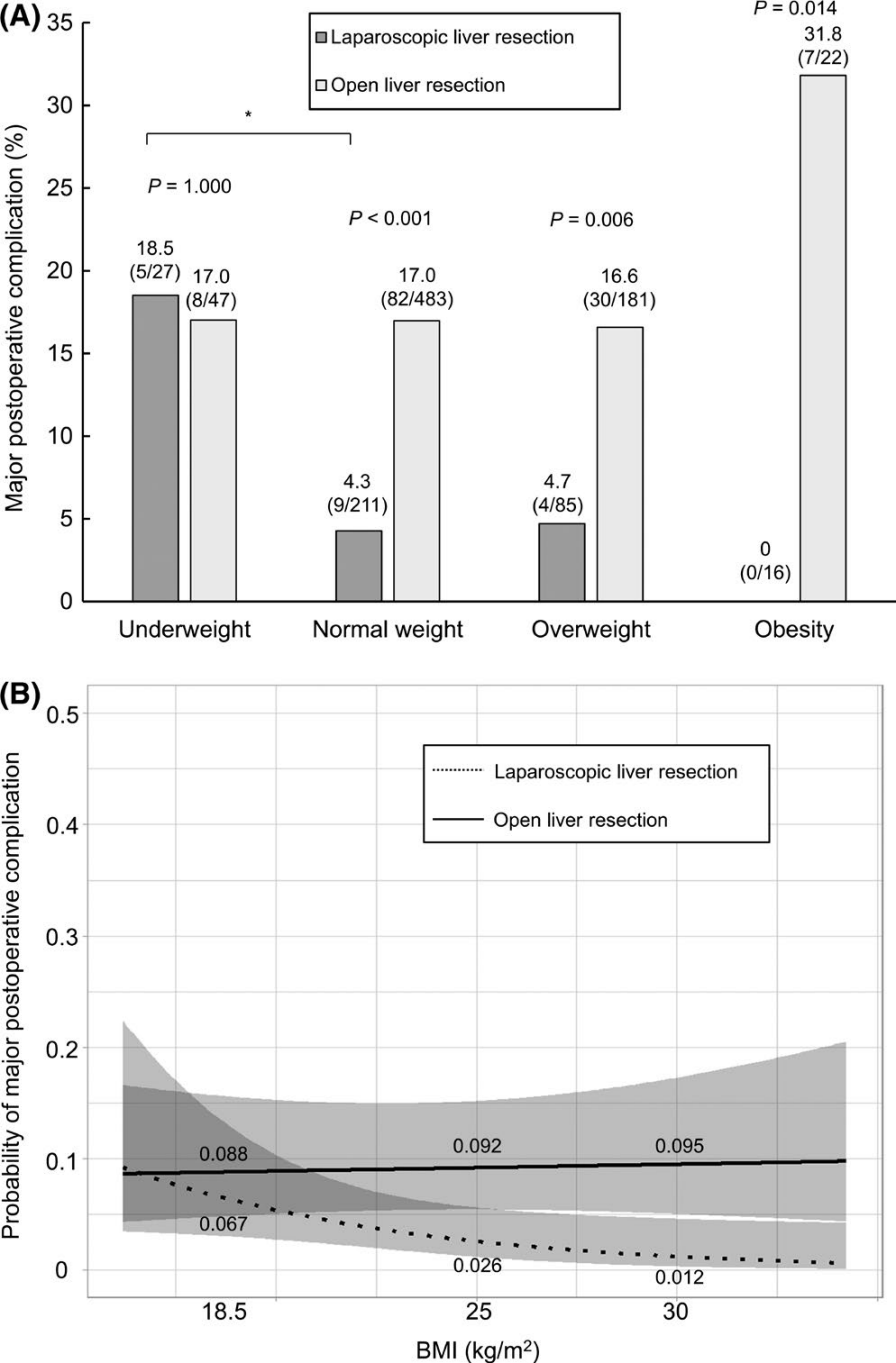
(A) Incidence of major postoperative complications according to BMI in patients who underwent LLR and OLR. The incidence of major postoperative complications had a stepwise decrease from underweight to obese patients in the LLR group (*P* = .013, Cochran‐Armitage trend test, *P* = .037), but not in the OLR group (*P* = .342). Underweight, BMI of ≤18.4 kg/m^2^; normal weight, 18.5 kg/m^2^ ≤ BMI ≤ 24.9 kg/m^2^; overweight, 25.0 kg/m^2^ ≤ BMI ≤ 29.9 kg/m^2^; and obesity, 30.0 kg/m^2^ ≤ BMI. BMI, body mass index; LLR, laparoscopic liver resection; OLR, open liver resection. *P < .01 using the Holm's method. (B) Risk of major postoperative complications after LLR and OLR according to BMI after adjusting for confounding variables via a multivariate analysis. A linear line with area represents the risk (mean with 95% confidence intervals). There was no difference in terms of the risk of major postoperative complications in patients with a BMI of 18.5 kg/m^2^. However, among patients with a BMI of 25.0 and 30.0 kg/m^2^, the risk was lower in the LLR group than in the OLR group. In addition, there was significant effect of interaction between patients who underwent LLR and those who underwent OLR according to increase in BMI value (*P* for interaction = .048). BMI, body mass index; LLR, laparoscopic liver resection; OLR, open liver resection. Major complication, ≥grade IIIa based on the Clavien‐Dindo classification system

The incidence of major complications after LLR indicated a stepwise decrease from the underweight to obese groups (Cochran‐Armitage trend test, *P* = .037, Figure 1A). A multiple analysis using Holm's test indicated that the incidence in the underweight group was higher than that in the normal weight group; however, there were no significant differences among the normal weight, overweight, and obese groups. The incidence of wound infection and intra‐abdominal infection did not differ among the four groups, and none of the patients presented with complex venous thromboembolism (Table 3). There was no significant difference in the incidence of each complication among the four BMI groups with zero in‐hospital death. In patients who underwent OLR, there was no difference in the incidence of overall or major complications among the four groups (Figure 1A). The incidence of wound infection and abdominal infection did not differ among the four groups, and only one (0.6%) patient in the overweight group had complex venous thromboembolism. One patient in the underweight group and three in the normal weight group died of liver failure. In the normal weight, overweight, and obesity groups, the incidence of major complications was higher in patients who underwent OLR than in those who underwent LLR (underweight group, *P* = .871; normal weight group, *P* < .001; overweight group, *P* = .006; and obesity group, *P* < .001).

**TABLE 3:** Postoperative course in patients with hepatocellular carcinoma (n = 1072) after laparoscopic or open liver resection according to body mass index

Variables	Laparoscopic liver resection group					Open liver resection group				
	Underweight group	Normal weight group	Overweight group	Obese group	*P* value	Underweight group	Normal weight group	Overweight group	Obese group	*P* value
	(n = 27)	(n = 211)	(n = 85)	(n = 16)		(n = 47)	(n = 483)	(n = 181)	(n = 22)	
Postoperative complications										
Overall	7 (25.9)	35 (16.6)	16 (18.8)	1 (6.3)	.397	17 (36.2)	179 (37.1)	64 (35.4)	10 (45.5)	.828
Wound infection	0	1 (0.5)	0	0	.894	2 (4.3)	22 (4.6)	8 (4.4)	1 (4.5)	1.000
Intra‐abdominal infection^†^	2 (7.4)	2 (0.9)	1 (1.2)	0	.065	4 (8.5)	38 (7.9)	12 (6.6)	1 (4.5)	.888
Bile leakage^†^	2 (7.4)	3 (1.4)	3 (3.5)	0	.197	3 (6.4)	30 (6.2)	8 (4.4)	4 (18.2)	.091
Ascites^†^	0	1 (0.5)	0	0	.894	0	21 (4.3)	3 (1.7)	1 (4.5)	.193
Pleural effusion^†^	1 (3.7)	1 (0.5)	2 (2.4)	0	.312	2 (4.3)	27 (5.6)	8 (4.4)	2 (9.1)	.780
Bleeding^†^	1 (3.7)	1 (0.5)	0	0	.164	1 (2.1)	2 (0.4)	0	0	.237
Gastrointestinal bleeding^†^	0	2 (0.9)	0	0	.748	0	2 (0.4)	1 (0.6)	1 (4.5)	.075
Respiratory complications^†^	0	2 (0.9)	0	0	.748	2 (4.3)	8 (1.7)	0	0	.110
Liver failure^‡^	0	1 (0.5)	0	0	.894	1 (2.1)	9 (1.9)	1 (0.6)	0	.574
Grade (A/B/C)	0/0/0	0/1/0	0/0/0	0/0/0		0/0/1	1/3/5	0/0/1	0/0/0	
Ileus^†^	0	0	1 (1.2)	0	.392	0	1 (0.2)	0	0	.915
Others^†^	1 (3.7)	1 (0.5)	0	0	.164	1 (2.1)	5 (1.0)	1 (0.6)	0	.744
In‐hospital death	0	0 0	0	‐		1 (2.1)	3 (0.6)	0	0	.343
Hospital stay, days; median (range)	10 (6‐88)	9 (4‐79)	9 (5‐86)	8 (6‐17)	.366	16 (6‐67)	15 (6‐212)	15 (5‐102)	14 (6‐134)	.901

^†^≥ Grade IIIa based on the Clavien‐Dindo classification. ^‡^International Study Group of Liver Surgery definition.

### 3.4 Risk factors for major complications after LLR and OLR

In all the cohorts including the LLR and the OLR groups, univariate and multivariate logistic regression analyses showed that age, operative time, volume of blood loss, serum albumin concentration, and LLR were independent predictive factors for major postoperative complications (Table S1). Meanwhile, BMI was not associated with major postoperative complications in all the cohorts including the LLR and OLR groups. However, in the LLR group, an increased BMI was considered an independent favorable factor and long operative time was a risk factor for postoperative complications (Table 4). On the contrary, among patients who underwent OLR, BMI was not a risk for postoperative major complication; however, long operative time was a risk factor.

**TABLE 4:** Univariate and multivariate analyses of risk factors associated with major complications after laparoscopic and open liver resection

Variables	Laparoscopic liver resection group				Open liver resection group			
	Univariate analysis		Multivariate analysis		Univariate analysis		Multivariate analysis	
	*P* value	Odds ratio (95% CI)	*P* value	Odds ratio (95% CI)	*P* value	Odds ratio (95% CI)	*P* value	Odds ratio (95% CI)
BMI, per 1 kg/m^2^ increase	.044	0.87 (0.75‐0.996)	.026	0.84 (0.73‐0.98)	.670	1.01 (0.96‐1.07)	.845	1.01 (0.95‐1.07)
Age, per 1 year increase	.220	1.04 (0.98‐1.09)	.258	1.04 (0.73‐1.53)	.342	1.01 (0.99‐1.03)	.072	1.02 (0.998‐1.05)
Sex, male/female	.259	2.07 (0.59‐7.32)	.519	1.59 (0.39‐6.48)	.097	1.57 (0.92‐2.69)	.207	1.45 (0.82‐2.58)
Comorbidities and/or previous history								
Diabetes mellitus	.474	0.68 (0.24‐1.96)			.055	1.48 (0.99‐2.20)		
Hypertension	.728	0.85 (0.33‐2.18)			.464	1.15 (0.79‐1.69)		
Dyslipidemia	.645	1.28 (0.44‐3.72)			.384	0.78 (0.45‐1.36)		
Ischemic heart diseases	.275	0.32 (0.04‐2.49)			.036	2.20 (1.06‐4.60)	.149	1.81 (0.81‐4.06)
Alcohol abuse	.359	0.59 (0.19‐1.83)			.283	1.25 (0.83‐1.89)		
Viral hepatitis	.880	0.92 (0.32‐2.66)			.178	0.76 (0.51‐1.13)		
Pathologically confirmed cirrhosis	.324	0.53 (0.15‐1.87)			.111	1.38 (0.93‐2.06)		
Laboratory tests								
Total bilirubin per 1 g/dL increase	.437	0.55 (0.12‐2.51)			.638	0.88 (0.51‐1.52)		
Albumin per 1 g/dL increase	.269	0.56 (0.20‐1.56)			.021	0.60 (0.38‐0.92)	.151	0.68 (0.40‐1.15)
Prothrombin activity per 1% increase	.188	1.02 (0.99‐1.05)			.039	0.99 (0.97‐0.99)	.267	0.99 (0.98‐1.01)
Child‐Pugh score, B to A	.999	‐ ‐			.030	2.65 (1.10‐6.39)	.467	1.51 (0.50‐4.61)
Platelet count per 1 × 10^4^/mL increase	.931	0.996 (0.92‐1.08)			.054	1.03 (1.00‐1.06)		
AST per 1 IU/L increase	.156	1.01 (0.996‐1.03)			.588	1.002 (0.995‐1.01)		
ALT per 1 IU/L increase	.933	1.001 (0.98‐1.02)			.888	1.00 (0.99‐1.01)		
Surgery‐related factors								
Recurrence	.607	1.30 (0.48‐3.58)			.578	1.13 (0.73‐1.75)		
Repeat liver resection	.837	0.85 (0.19‐3.85)			.705	0.90 (0.51‐1.57)		
Sectionectomy or more	.003	4.47 (1.64‐12.20)	0.707	1.31 (0.32‐5.46)	<.001	2.16 (1.46‐3.18)	0.549	1.17 (0.70‐1.95)
Operative time per 1 h increase	<.001	1.70 (1.37‐2.12)	0.011	1.47 (1.09‐1.99)	<.001	1.34 (1.23‐1.46)	<.001	1.27 (1.11‐1.46)
Bleeding per 1 mL increase	<.001	4.21 (2.05‐8.63)	0.125	2.22 (0.80‐6.12)	<.001	1.59 (1.36‐1.86)	.071	1.23 (0.98‐1.53)
Non‐curative surgery	.999	‐	‐		.583	0.71 (0.21‐2.42)		
Tumor‐related factors								
AFP level (≥ 20 ng/mL)	.978	0.986 (0.36‐2.70)			0.638	1.10 (0.75‐1.62)		
Tumor size per 1 cm increase	.003	1.47 (1.14‐1.89)	.753	1.06 (0.73‐1.53)	.016	1.07 (1.01‐1.14)	.091	0.93 (0.86‐1.01)
Number, multiple to solitary	.590	0.66 (0.15‐2.97)			.716	1.08 (0.71‐1.63)		
Macrovascular invasion	.999	‐			.017	1.82 (1.11‐2.99)	.749	1.12 (0.57‐2.20)
UICC stage per 1 stage increase	.179	1.48 (0.84‐2.62)			.011	1.24 (1.05‐1.46)	.834	0.98 (0.77‐1.32)
Pathology								
Poor HCC	.901	1.08 (0.30‐3.88)			.656	0.91 (0.59‐1.39)		
Number, multiple to solitary	.380	0.51 (0.12‐2.28)			.988	1.003 (0.67‐1.51)		
Microvascular invasion	.093	2.32 (0.87‐6.22)			.571	1.12 (0.76‐1.67)		

Abbreviations: AFP, alpha fetoprotein; AST, aspartate aminotransferase; ALT, alanine aminotransferase; BMI, body mass index; CI, confidence interval; COPD, chronic obstructive pulmonary disease; HBV, hepatitis B virus; HCC, hepatocellular carcinoma; HCV, hepatitis C virus; UICC, Union for International Cancer Control.

After adjusting for age, sex, liver function test, and surgery‐ and tumor‐related factors between the LLR and OLR groups, the risk of major postoperative complications in the LLR group decreased with increased BMI, while that in the OLR group increased with greater BMI (Figure 1B). In patients with a BMI of 25 and 30, but not 18.5, kg/m^2^, the risk was lower in the LLR group than in the OLR group. In addition, there was significant effect of interaction between patients who underwent LLR and those who underwent OLR according to increase in BMI value (*P* for interaction = .048, Figure 1B).

## 4 DISCUSSION

Countermeasures for the depth of surgical field and large volume of intraperitoneal fat are important in abdominal surgery, including liver resection, in overweight and obese patients.^33^, ^34^ In this study, a high BMI was associated with operative time and volume of blood loss in the OLR group, but not in the LLR group. Despite a large skin incision and compression of the gastrointestinal tract and greater omentum in OLR, liver parenchyma dissection and treatment of hepatic hilum are sometimes challenging, and this can be associated with long operative time and large volume of blood loss. This finding was in accordance with that of other reports.^9^, ^35^, ^36^, ^37^, ^38^ In contrast, in LLR, pneumoperitoneum, head up position, and high magnification—even at deep portions in the caudal view—can provide sufficient free space to control the forceps, even in overweight and obese patients.^39^, ^40^ These conditions cannot affect operative time or volume of blood loss in overweight and obese patients. Even after adjusting for confounding variables, the risk of major postoperative complications was higher in overweight and obese patients (BMI of 25 and 30 kg/m^2^, respectively) in the OLR group than in the LLR group. Based on these data, the physical characteristics of overweight and obese patients can be risk factors for major complications after OLR compared with LLR. We showed a stepwise decrease in the incidence of major postoperative complications according to increasing BMI. Hence, LLR should be recommended for eligible overweight and obese patients to prevent major postoperative complications. Obesity is a high‐risk factor for several complications such as infections, venous thromboembolism, and respiratory complications after OLR.^10^, ^41^, ^42^ In this study, there was no significant difference in terms of complications associated with OLR among the four BMI groups despite a greater proportion of comorbidities, including DM. This phenomenon can be attributed to controlling comorbidities before surgery and preventing surgical site infection.^43^, ^44^

Moreover, even after adjusting for confounding variables between the LLR and OLR groups, there was no difference in terms of the incidence of major complications among underweight patients in the LLR and OLR groups. Some studies have shown that the risk of postoperative complications after abdominal or non‐abdominal surgery according to BMI has a reverse J‐shape relationship, with underweight and morbidly obese individuals showing the highest rates and overweight and moderately obese individuals showing the lowest rates.^45^, ^46^, ^47^ Yu et al^33^ revealed that underweight patients who underwent LLR had a higher complication rate than normal body weight patients, which corresponded with our results. These phenomena are referred to as the obesity paradox, and its cause has not been fully elucidated. However, underweight‐induced hypoalbuminemia, sarcopenia, frailty, malnutrition, and a poor immune function may be correlated with poor outcomes after surgery.^48^, ^49^, ^50^, ^51^ In this study, the albumin level was basically good because the liver function reserve (e.g. the Child‐Pugh classification) was assessed before hepatectomy. In fact, there was no difference in albumin level among the BMI groups in patients who underwent LLR. In addition, we did not evaluate sarcopenia or frailty; thus, we could not elucidate the cause of the higher proportion of major complications in the underweight group after LLR in our own study. However, we could suggest that sufficient operative space could be obtained during LLR, even in overweight and obese patients, and that this was associated with surgical safety and a decreased incidence of major complications.

The current study had several limitations. That is, it has a retrospective design, and the number of patients was large. Moreover, the research was conducted at a single institution. A BMI of >40 kg/m^2^ was considered a risk factor for in‐hospital death.^52^ However, none of the patients had a BMI of >40 kg/m^2^. Further, LLR was safely performed on each eligible patient. LLR and OLR have different indications according to patient and tumor characteristics, both of which may act as independent risk factors for postoperative complications. Nevertheless, the results of this study should be validated by performing multicenter and international studies.

## 5 CONCLUSION

Laparoscopic liver resection is superior to OLR in overweight and obese patients in regard to decrease in incidences of postoperative major complications.

## DISCLOSURE

### Funding

No funding was received for this study.

### Conflict of Interest

Authors declare no conflict of interests for this article.

### Disclosure of Ethical Statements

This research was approved by the Ethics Committee of the institution (Committee of Osaka City University Graduate School of Medicine, Approval No. 1646) and it conforms to the provisions of the Declaration of Helsinki. Informed consent was obtained from all the subjects and/or guardians.

### Author Contributions

Project development: A. Ishihara, S. Tanaka, H. Shinkawa, and S. Kubo; Acquisition of data: A. Ishihara, S. Tanaka, H. Shinkawa, H. Yoshida, S. Takemura, R. Amano, K. Kimura, G. Ohira, and K. Nishio. Analysis and interpretation of data: A. Ishihara, S. Tanaka, H. Shinkawa, H. Yoshida, S. Kubo. Drafting of the manuscript: A. Ishihara and S. Tanaka. Editing of the manuscript: All authors. All authors have approved the final manuscript and agree to be accountable for all aspects of the work.

## Supporting information

Table S1
