# Supplementary material for: Superiority of laparoscopic liver resection to open liver resection in obese individuals with hepatocellular carcinoma: A retrospective study
Source: Ann Gastroenterol Surg. 2021 Sep 16;6(1):135–48. doi: 10.1002/ags3.12506 (PMC8786695; doi:10.1002/ags3.12506)
Supplement: Supplementary file 1 — Table S1 [file AGS3-6-135-s001.docx]

**Supplemental Table 1.** Univariate and multivariate analyses of risk factors associated with major complications after liver resection for hepatocellular carcinoma

|  | Univariate analysis | | |  | Multivariate analysis | | |
| --- | --- | --- | --- | --- | --- | --- | --- |
| Variables | *P* value | Odds ratio | (95% CI) |  | *P* value | Odds ratio | (95% CI) |
| BMI per 1 kg/m^2^ increase | 0.458 | 0.98 | (0.93–1.03) |  | 0.376 | 0.98 | (0.92–1.03) |
| Laparoscopic approach | < 0.001 | 0.27 | (0.16–0.45) |  | 0.001 | 0.39 | (0.22–0.68) |
|  |  |  |  |  |  |  |  |
| Age per 1 year increase | 0.194 | 1.01 | (0.99–1.03) |  | 0.043 | 1.02 | (1.001–1.05) |
| Sex, male/female | 0.015 | 1.83 | (1.12–2.97) |  | 0.174 | 1.44 | (0.85–2.44) |
| Comorbidities and/or previous history |  |  |  |  |  |  |  |
| Diabetes mellitus | 0.215 | 1.26 | (0.88–1.81) |  |  |  |  |
| Hypertension | 0.860 | 1.03 | (0.73–1.47) |  |  |  |  |
| Dyslipidemia | 0.298 | 0.77 | (0.48–1.25) |  |  |  |  |
| Ischemic heart diseases | 0.036 | 2.05 | (1.05–4.02) |  | 0.095 | 1.89 | (0.90–3.97) |
| Alcohol abuse | 0.678 | 1.08 | (0.74–1.58) |  |  |  |  |
| Viral hepatitis | 0.126 | 0.75 | (0.52–1.09) |  |  |  |  |
| Pathologically confirmed cirrhosis | 0.164 | 1.30 | (0.90–1.88) |  |  |  |  |
|  |  |  |  |  |  |  |  |
| Laboratory tests |  |  |  |  |  |  |  |
| Total bilirubin level per 1 g/dL increase | 0.754 | 0.93 | (0.57–1.51) |  |  |  |  |
| Albumin level per 1 g/dL increase | 0.001 | 0.52 | (0.35–0.76) |  | 0.012 | 0.56 | (0.35–0.88) |
| Prothrombin activity per 1% increase | 0.141 | 0.99 | (0.98–1.00) |  |  |  |  |
| Child–Pugh score, B to A | 0.148 | 0.55 | (0.25–1.24) |  |  |  |  |
| Platelet count, per 1×10^4^/mL increase | 0.023 | 1.03 | (1.004–1.06) |  | 0.507 | 1.01 | (0.98–1.04) |
| AST level per 1 IU/L increase | 0.068 | 1.01 | (1.00–1.01) |  |  |  |  |
| ALT level per 1 IU/L increase | 0.465 | 1.00 | (0.996–1.01) |  |  |  |  |
|  |  |  |  |  |  |  |  |
| Surgery-related factors |  |  |  |  |  |  |  |
| Recurrence | 0.605 | 1.11 | (0.75–1.64) |  |  |  |  |
| Repeat liver resection | 0.768 | 0.92 | (0.55–1.55) |  |  |  |  |
| Sectionectomy | < 0.001 | 2.84 | (1.99–4.06) |  | 0.557 | 1.16 | (0.71–1.87) |
| Operative time per 1 h increase | < 0.001 | 1.42 | (1.31–1.54) |  | < 0.001 | 1.33 | (1.18–1.51) |
| Volume of blood loss per 1 mL increase | < 0.001 | 1.81 | (1.55–2.11) |  | 0.059 | 1.23 | (0.99–1.53) |
| Non-curative surgery | 0.822 | 0.87 | (0.26–2.94) |  |  |  |  |
|  |  |  |  |  |  |  |  |
| Tumor-related factors |  |  |  |  |  |  |  |
| AFP level (≥ 20 ng/mL) | 0.433 | 1.15 | (0.81–1.65) |  |  |  |  |
| Tumor size, per 1 cm increase | < 0.001 | 1.13 | (1.07–1.19) |  | 0.056 | 0.93 | (0.86–1.002) |
| Number, multiple to solitary | 0.313 | 1.22 | (0.83–1.80) |  |  |  |  |
| Macrovascular invasion | 0.001 | 2.23 | (1.40–3.55) |  |  |  |  |
| UICC stage, per 1 stage increase | < 0.001 | 1.38 | (1.18–1.61) |  | 0.949 | 0.994 | (0.82–1.20) |
| Pathology |  |  |  |  |  |  |  |
| Poor HCC | 0.697 | 1.08 | (0.73–1.61) |  |  |  |  |
| Number, multiple to solitary | 0.625 | 1.10 | (0.75–1.61) |  |  |  |  |
| Microvascular invasion | 0.071 | 1.40 | (0.97–2.01) |  |  |  |  |

AFP, alpha fetoprotein; AST, aspartate aminotransferase; ALT, alanine aminotransferase; BMI, body mass index; COPD, chronic obstructive pulmonary disease; HBV, hepatitis B virus; HCC, hepatocellular carcinoma; HCV, hepatitis C virus; UICC, Union for International Cancer Control
